# Spontaneous dissection of the coronary and vertebral arteries post-partum: case report and review of the literature

**DOI:** 10.1186/1471-2393-12-122

**Published:** 2012-11-02

**Authors:** Marta Cenkowski, Megan daSilva, Kimberly-Ann Bordun, Farrukh Hussain, Iain DC Kirkpatrick, Davinder S Jassal

**Affiliations:** 1 Department of Anesthesia, University of Manitoba, Winnipeg, Manitoba, Canada; 2 Institute of Cardiovascular Sciences, St. Boniface General Hospital, University of Manitoba, Winnipeg, Manitoba, Canada; 3 Section of Cardiology, Department of Internal Medicine, University of Manitoba, Winnipeg, Manitoba, Canada; 4 Department of Radiology, University of Manitoba, Winnipeg, Manitoba, Canada; 5 Cardiology, Radiology and Physiology Rm Y3531, Bergen Cardiac Care Centre Section of Cardiology, Department of Internal Medicine Faculty of Medicine, University of Manitoba St. Boniface General Hospital, 409 Taché Avenue, Winnipeg, Manitoba, R2H 2A6, Canada

**Keywords:** Spontaneous dissection, Vasculature, Pregnancy, Imaging

## Abstract

**Background:**

Spontaneous coronary and vertebral artery dissections are rare events occurring most commonly in otherwise healthy women during pregnancy or the post-partum period.

**Case presentation:**

This report describes a 35-year-old female who presented with an acute inferior ST elevation myocardial infarction 7 months post-partum secondary to spontaneous dissection of the left obtuse marginal coronary artery. Despite appropriate medical therapy with dual anti-platelet therapy, the patient presented four weeks later with a spontaneous dissection of the right vertebral artery.

**Conclusion:**

We review the presentation, diagnosis, and management of spontaneous dissections of the vasculature in the peri-partum period.

## Background

Spontaneous coronary and vertebral artery dissections are rare events occurring most commonly in otherwise healthy women during pregnancy or the post-partum period.

## Case presentation

**Figure 1:**
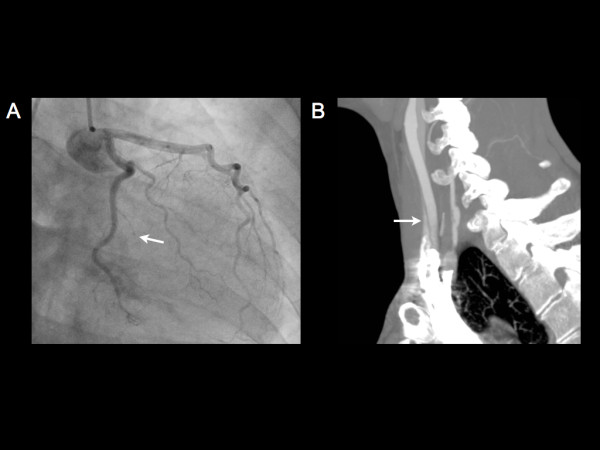
A) Diffuse tapering and occlusion in the mid body of the obtuse marginal 2 consistent with a dissection and occlusive intramural hematoma; B) Sagittal oblique maximum intensity projection reformation of a CT angiogram of the carotid and vertebral arteries demonstrates a focal dissection with associated localized aneurysmal dilatation of the V1 segment of the right vertebral artery.

A 35 year old G2, P2 female presented with a two hour history of retrosternal chest discomfort radiating to the jaw, associated with nausea and diaphoresis. She was 7 months post-partum and had no underlying cardiovascular risk factors. On physical examination, the heart rate was 84 bpm with a blood pressure of 115/70 mm Hg. The jugular venous pressure, heart sounds and breath sounds were within normal limits. The initial EKG demonstrated evidence of an acute inferior ST elevation myocardial infarction (STEMI). On coronary angiography, there was evidence of a distal dissection of the left second obtuse marginal (OM2) coronary artery, with no percutaneous options for repair (Figure 1A). During the patient’s admission to CCU, the cardiac enzyme levels including creatine kinase and high sensitivity troponin T peaked at 1340 U/L and 3702 ng/L, respectively. Transthoracic echocardiography (TTE) demonstrated mild hypokinesis of the basal inferior wall with a left ventricular ejection fraction of 50-55%. As the hematologic and connective tissue disease work-up was negative, the patient was diagnosed with an acute inferior STEMI secondary to spontaneous dissection of the OM2. The patient was appropriately discharged on dual antiplatelet therapy including ASA and Clopidogrel including beta blockade with Metoprolol.

One month later, the patient returned with diplopia and numbness of her left arm and face. Computed tomographic angiography demonstrated a focal 8 mm long dissection of the right vertebral artery in its V1 segment with mild associated aneurysmal dilatation (Figure 1B). No other abnormalities were present in the angiogram and repeat TTE was within normal limits. With the diagnosis of spontaneous dissection in two separate vascular territories, including the coronary and vertebral arteries, the patient was investigated with genetic testing to rule out any underlying collagen disorders including Ehlers Danlos and Marfan syndrome, which was within normal limits. The patient was subsequently started on anticoagulation therapy to prevent future spontaneous dissections.

## Conclusion

Spontaneous coronary and vertebral artery dissections are rare, life-threatening events occurring most commonly in otherwise healthy women during pregnancy or the post-partum period [1,2]. In this patient population, spontaneous coronary artery dissection (CAD) is the cause of approximately 16% of myocardial infarctions (MI), as compared to only 1% in the general population [3]. A more rare occurrence is spontaneous vertebral artery dissection (VAD) which has an incidence of only 0.0015% in the pregnant population [3]. Spontaneous arterial dissection results from rupture and separation of the media creating a false lumen within the vessel wall without a traumatic or iatrogenic cause. Passage of blood into this false lumen forces the intimal-medial layer towards the true lumen of the vessel, causing partial or full obstruction of flow, ultimately leading to an ischemic event [1].

The pathogenesis of spontaneous vascular dissection is unclear [1,3]. Spontaneous dissection of the vasculature may be associated with pregnancy, oral contraceptive use, cocaine use, or excessive exercise [1,4]. Spontaneous CAD and VAD can also be the result of minor blunt force trauma, normally in combination with predisposing factors including collagen disorders such as Ehlers Danlos and Marfan syndrome [1-3]. Physiological changes of pregnancy have also been proposed as potential mechanisms of injury to the vessel wall [3,4]. High levels of progesterone or decreased collagen synthesis during pregnancy may weaken the tunica media of the vessel walls [3,4]. As cardiac output increases by approximately 50% during pregnancy, the increase in shear stress on the vessel wall can cause a greater risk of media rupture [3].

The clinical presentation of dissection of the coronary or vertebral arteries varies from patient to patient. In women with spontaneous CAD, although chest pain is the most common presenting symptom, they may present with a myocardial infarction, cardiogenic shock or sudden cardiac death [1]. Spontaneous VAD can present as headache, neck pain, diplopia, transient ischemic attack, Horner’s syndrome, or an ischemic stroke [2].

Although invasive coronary angiography is routinely used to diagnose spontaneous CAD, cardiac computed tomography (CCT) can provide complementary information [1]. On coronary angiography, a dissection will present as contrast media within two layers separated by an intimal flap with delayed clearance of contrast within the false lumen [1,5]. When these findings are observed in otherwise healthy young patients presenting with an acute coronary syndrome, spontaneous CAD should be suspected, and confirmed by intravascular ultrasound [1,5]. Although CCT may allow for the early detection of SCAD if angiography is not readily available, it is an excellent imaging test for follow-up of these lesions [1]. In a patient presenting with neurological symptoms suggestive of a spontaneous VAD, either CT or MRI angiography will be able to accurately visualize the vessel lumen and confirm the diagnosis [2].

The treatment for spontaneous CAD and VAD is multifaceted including either conservative medical therapy and/or invasive management with PCI or CABG. Antiplatelet therapy and anti-coagulants are often used to prevent future ischemic events, allowing the dissection to heal on its own [2,3]. In the case of spontaneous CAD, percutaneous coronary intervention (PCI) with stenting has been shown to be an effective management technique as well as coronary artery bypass grafting (CABG). In a retrospective analysis of all patients who presented with spontaneous CAD, intervention with PCI or CABG were reported in up to 50% of the population [5]. Spontaneous VAD are usually treated with anticoagulation therapy for a minimum of 3–6 months [2,3].

In the literature, there are only two previous case reports describing pregnant women with both coronary and vertebral artery dissections (Table 1). The first case describes a 33 year old G2P2 woman who presented three days post-partum with a dissection of the right coronary artery who underwent PCI and stenting. Two days following her spontaneous CAD, she was found to have a VAD and was treated medically with a favorable result [4]. The second report describes a 28 year old G2P2 woman who presents 10 days post-partum with chest pain and headache and was found to have simultaneous spontaneous CAD and VAD (Table 1). She underwent CABG and was medically treated with antiplatelet and anticoagulation therapy [3]. In our patient, despite appropriate medical treatment with dual anti-platelet therapy and aggressive beta blockade for spontaneous dissection of the OM2, she developed a spontaneous VAD one month later requiring the addition of anticoagulation therapy as well. All three cases illustrate how a high index of suspicion is necessary for the appropriate diagnosis and management of spontaneous CAD and/or VAD in the pregnant patient population. 

**Table 1:** Summary of findings in post-partum spontaneous CAD and VAD cases

**Case**	**ID**	**Presentation**	**Imaging**	**Treatment/ Follow-up**
Motreff et al. (2010)	33 F G2P2	2 days post partum	● ECHO: LVEF 58%	● PCI with stent of RCA
		● ACS 5 days post partum		● Medical management for VAD
		● Vertebral artery dissection		
Sharma et al. (2010)	28 F G2P2	10 days post partum	● Coronary CT Angiography: soft plaque in the LAD	● CABG involving LIMA to LAD and saphenous vein bypass graft to OM1
		● Non-exertional, intermittent, sub-sternal, sharp chest pain, and left arm numbness	● Coronary angiogram: 90% eccentric stenosis in the ostial LAD extending to first septal perforator and dissection of the LCx	● In OR: Healed dissection in LCx and fresh dissection in LAD
		● Intermittent bi-frontal headache	● IVUS: dissection flap in LAD with flow only in true lumen and intraluminal filling defect along entire course of LCx through AV groove	● Medical therapy: ASA Metoprolol, Simvastatin, Coumadin x 6 weeks
		● EKG STEMI V1-V2	● MRA cervicocephalic vessels: 50% stenosis with a double lumen in mid-cervical left vertebral artery consistent with dissection	● MRA (6 weeks later): healed vertebral artery dissection and clinically free of symptoms
		● CK and Troponin elevated		
Cenkowski et al. (2012)	35 F G2P2	7 months post partum	● EKG: Inferior STEMI	7 months post partum
		● Sudden onset retrosternal chest pain radiating to jaw, nausea, vomiting	● Coronary angiography: distal dissection of OM2	● Coronary artery dissection distal and not amenable to percutaneous repair
		8 months post partum	● Transthoracic echo: mild hypokinesis basal inferior wall and EF 50-55%	● Medical therapy: ASA, Clopidogrel, Metoprolol, Ramipril, Simvastatin
		● Diplopia, numbness to left arm and face	● CT angiography: 8mm dissection R vertebral artery in its V1 segment	● Medical therapy: Warfarin x 6 months in addition to ASA and Clopidogrel

STEMI, ST elevation myocardial infarction; EF, ejection fraction; LAD, left anterior descending coronary artery; LCx, left circumflex coronary artery; OM2, second obtuse marginal branch; OM1, first obtuse marginal branch; CABG, coronary artery bypass graft; CT, computed tomography; LIMA, left internal mammary artery.

## Consent

Written informed consent was obtained from the patient for publication of this case report and accompanying images. A copy of the written consent is available for review by the editor of this journal.

## Competing interests

The authors declare that they have no competing interests.

## Authors’ contributions

All authors contributed to the writing of the manuscript. All authors read and approved the final manuscript.

## Pre-publication history

The pre-publication history for this paper can be accessed here:

http://www.biomedcentral.com/1471-2393/12/122/prepub
